# CD4^+^CD38^+^ central memory T cells contribute to HIV persistence in HIV-infected individuals on long-term ART

**DOI:** 10.1186/s12967-020-02245-8

**Published:** 2020-02-24

**Authors:** Cheng-Bo Song, Le-Le Zhang, Xian Wu, Ya-Jing Fu, Yong-Jun Jiang, Hong Shang, Zi-Ning Zhang

**Affiliations:** 1 NHC Key Laboratory of AIDS Immunology (China Medical University), Department of Laboratory Medicine, The First Affiliated Hospital of China Medical University, No 155, Nanjingbei Street, Heping District, Shenyang, 110001 Liaoning Province China; 2 National Clinical Research Center for Laboratory Medicine, The First Affiliated Hospital of China Medical University, Shenyang, 110001 China; 3 Key Laboratory of AIDS Immunology of Liaoning Province, The First Affiliated Hospital of China Medical University, Shenyang, 110001 China; 4 Key Laboratory of AIDS Immunology, Chinese Academy of Medical Sciences, Shenyang, 110001 China; 5 Collaborative Innovation Center for Diagnosis and Treatment of Infectious Diseases, 79 Qingchun Street, Hangzhou, 310003 China

**Keywords:** HIV, Reservoir, CD38, Tcm, CD4^+^ T cell

## Abstract

**Background:**

Despite the effective antiretroviral treatment (ART) of HIV-infected individuals, HIV persists in a small pool. Central memory CD4^+^ T cells (Tcm) make a major contribution to HIV persistence. We found that unlike HLA-DR, CD38 is highly expressed on the Tcm of HIV-infected subjects receiving ART for > 5 years. It has been reported that the half-life of total and episomal HIV DNA in the CD4^+^CD38^+^ T cell subset, exhibits lower decay rates at 12 weeks of ART. Whether CD38 contributes to HIV latency in HIV-infected individuals receiving long-term ART is yet to be addressed.

**Methods:**

Peripheral blood mononuclear cells (PBMCs) were isolated from the whole blood of HIV-infected subjects receiving suppressive ART. The immunophenotyping, proliferation and apoptosis of CD4^+^ T cell subpopulations were detected by flow cytometry, and the level of CD38 mRNA and total HIV DNA were measured using real-time PCR and digital droplet PCR, respectively. A negative binomial regression model was used to determine the correlation between CD4^+^CD38^+^ Tcm and total HIV DNA in CD4^+^ T cells.

**Results:**

CD38 was highly expressed on CD4^+^ Tcm cells from HIV infected individuals on long-term ART. Comparing with HLA-DR^−^Tcm and CD4^+^HLA-DR^+^ T cells, CD4^+^CD38^+^ Tcm cells displayed lower levels of activation (CD25 and CD69) and higher levels of CD127 expression. The proportion of CD38^+^ Tcm, but not CD38^−^ Tcm cells can predict the total HIV DNA in the CD4^+^ T cells and the CD38^+^ Tcm subset harbored higher total HIV DNA copy numbers than the CD38^−^ Tcm subset. After transfected with CD38 si-RNA in CD4^+^ T cells, the proliferation of CD4^+^ T cells was inhibited.

**Conclusion:**

The current date indicates that CD4^+^CD38^+^ Tcm cells contribute to HIV persistence in HIV-infected individuals on long-term ART. Our study provides a potential target to resolve HIV persistence.

## Background

Antiretroviral therapy (ART) induces durable suppression of plasma viremia and prolongs the lifespan of HIV-infected patients [1, 2]. However, the persistence of HIV reservoirs remains a barrier to the resolution of HIV disease in infected individuals receiving suppressive ART [3–5]. Once ART is discontinued, sustained virological remission cannot be achieved [6]. HIV establishes persistent infection in a number of cell types, localized to different anatomical compartments, via diverse mechanisms [1, 7, 8]. Understanding the mechanism of HIV persistence in the context of ART is critical for developing novel strategies targeting residual viral reservoirs.

Various cells are involved in the establishment and maintenance of the reservoir. Due to its relatively large size, retention of proliferative ability, and long life span, the central memory T (Tcm) cell subset is one of the most significant HIV reservoirs [9–11]. In HIV infection, HLA-DR and CD38 are well characterized markers of immune activation [12]. A 1997 study found that the expression of CD38 on CD8^+^ T cells correlated with the development of AIDS [12, 13], and has since been confirmed as a marker of HIV disease progression [14–16]. Although CD38 expression on CD4^+^ T cells is also related to immune activation, a study examining children infected with HIV during the perinatal period (with > 5 year survival), has shown that unlike its expression on CD8^+^ T cells, CD38 expression on CD4^+^ T cells may instead define a subset of immature cells [17]. Thus, CD38 is likely to perform a different function when expressed on CD4^+^ versus CD8^+^ T cells. Our analysis of the expression of CD38 and HLA-DR on T cells, revealed that, unlike HLA-DR, CD38 is highly expressed on CD4^+^ naive T cells (Tn) and CD4^+^ Tcm cells. In line with our findings, high CD38 expression levels have also been reported in the CD4^+^ Tcm cell subset of patients with B cell chronic lymphocytic leukemia (CLL) [18]. This raises the question, regarding the role of CD38, other than activation marker, when expressed on CD4^+^ Tcm cells in the context of HIV infection.

Besides its well-known character as an activation marker, the nature of CD38 is a circular ADP ribose hydrolase, which can catalyze the conversion of NAD [19]. Because of this activity, CD38 knockdown in mice enhances the anti-tumor ability of T cells via the NAD-SIRT1-FOXO1 axis [20]. It has been reported that activation of CD38 signaling, via an agonistic monoclonal antibody, prevents the apoptosis of human germinal center B cells [21]. In addition, CD38/CD31 interactions activate the genetic pathways leading to the proliferation of CLL cells [22]. CD38 expression may thus prolong the proliferation and survival of CD4^+^ Tcm cells, the major sites for the HIV reservoir, contributing to HIV latency and supporting HIV persistence [11]. Because CD38 expression is high in Tcm, which are the main population of HIV reservoir, these studies raised the question about whether CD38 supports HIV persistence. Previous studies had indicated the possibility that expression of CD38 molecule related with HIV reservoir. CD4^+^ T cells expressing PD-1, TIGIT, and LAG-3, alone or in combination, are associated with HIV persistence during ART [23–25], with the expression of PD-1 and LAG-3 being higher on CD4^+^CD38^+^ T cells [26]. Long-term ART typically shortens the half-life of HIV DNA and the clearance of HIV reservoirs [27–29]. Furthermore, it has been reported that the half-life of total and episomal HIV DNA in the CD4^+^CD38^+^ T cell subset, exhibits lower decay rates after 12 weeks of ART [30]. Whether CD38 contributes to HIV latency in HIV-infected individuals receiving long-term ART is yet to be addressed.

In this study, we recruited HIV-infected subjects under suppressive ART for at least 5 years. We found that the expression of CD38 on CD4^+^ Tcm cells was significantly higher than that of HLA-DR and that the expression of CD25, CD69, and CD127 on these CD38^+^ Tcm cells was similar to the classic HIV reservoir cells. Furthermore, we found that the proportion of CD4^+^CD38^+^ Tcm cells is effective in predicting total HIV DNA in CD4^+^ T cells, with CD38^+^ cells contributing more to HIV persistence than CD38^−^ cells by promoting proliferation. Therefore, our work demonstrates that CD4^+^CD38^+^ Tcm cells contribute to HIV persistence in HIV-infected individuals receiving long-term ART.

## Materials and methods

### Patient selection

For the purpose of this study, 36 HIV-infected participants receiving suppressive ART were enrolled at the First Hospital of China Medical University. Participants had been receiving suppressive ART, had > 350 cells/μl CD4^+^ T cell counts, and < 50 copies/ml HIV RNA. The ethical review committee from the First Hospital of China Medical University approved the collection of blood samples from HIV-infected individuals and written informed consent for participation in the study was obtained from all patients.

### Immunophenotyping

Peripheral blood mononuclear cells (PBMCs) were isolated from whole blood by Ficoll centrifugation. The following monoclonal antibodies (mAbs) and reagents were used in this study: PE-Cy7-conjugated anti-CD3, APC-conjugated anti-CD3, APC-Cy7-conjugated anti-CD4, PE-conjugated anti-CD38, APC-conjugated anti-HLA-DR, APC-Cy7-conjugated anti-HLA-DR, FITC-conjugated anti-CD45RA, PerCP-Cy5.5-conjugated anti-CCR7 (BD Biosciences, USA); Violet-conjugated anti-CD38, FITC-conjugated anti-CD38, PE-Cy7 conjugated anti-CD25, APC conjugated anti-CD69, APC conjugated anti-CD127, Amcyan-conjugated anti-CD45RA (BioLegend, San Diego, CA, USA). For the expression of all markers, flow cytometric gating was defined using fluorescence minus one (FMO) controls. CD4^+^ T cell subsets were identified in terms of CD45RA and CCR7 expression. CD38 and HLA-DR were measured on gated CD4^+^ T cell subsets: naive CD4^+^ T cells (Tn, CD3^+^CD4^+^CD45RA^+^CCR7^+^), central memory CD4^+^ T cells (Tcm, CD3^+^CD4^+^CD45RA^−^CCR7^+^), and effector memory CD4^+^ T cells (Tem, CD3^+^CD4^+^CD45RA^−^CCR7^−^). The expression of CD25, CD69, and CD127 were measured on gated CD38^+^ Tcm, HLA-DR^−^ Tcm, and CD4^+^HLA-DR^+^ cells. Data were collected using a BD LSRII flow cytometer (BD Biosciences) and analyzed using Flowjo software (TreeStar, USA).

### Cell sorting

Total CD4^+^ T cells were isolated from PBMCs using magnetic depletion as per the manufacturer’s protocol (Stem Cell Technologies, Canada). To further isolate CD38^+^ Tcm and CD38-Tcm, PBMCs were stained with the following antibodies: CD3-PE-Cy7, CD4-APC-Cy7, CD38-PE, CD45RA-FITC, CCR7-Percp-CY5.5 (all from BD Biosciences). Cells were sorted using a FACS Aria flow cytometer (BD Biosciences).

### Assessment of cell-associated HIV-1 DNA

Total DNA was extracted from total CD4^+^ T cells and Tcm cells, collected from HIV-infected individuals, using the QIAamp blood DNA mini kit (Qiagen, Germany) according to the manufacturer’s protocol. Total CD4^+^ T cell- and Tcm-derived HIV DNA was amplified using digital droplet PCR (ddPCR) (Bio-Rad, USA) using the primers and probes described in Additional file 1: Table S1. PCR was performed using the following program: 95 °C for 10 min, 50 cycles of 94 °C for 30 s, and 60 °C for 1 min, 98 °C for 10 min, then cooling at 16 °C. The droplets were subsequently read using the QX100 droplet reader, and the data were analyzed using QuantaSoft software (Bio-Rad).

### CD38-siRNA delivery

Transfection of primary CD4^+^ T cells with CD38-siRNA was performed with RNAiMAX (Invitrogen, USA) according to the manufacturer’s protocol. The CD38 knockdown process was achieved by employing 20 µM CD38 siRNA for 48 h (Invitrogen). Non-specific Stealth RNAi^®^ Negative Control Duplexes (Invitrogen) served as a siRNA control.

### RNA extraction and quantitative real-time PCR

Total RNA was isolated after a 48-h CD38-siRNA transfection by RNeasy Micro kit (Qiagen, USA). Then the purified RNA was treated to eliminate genomic DNA contamination using DNase I reagent. The RNA was reversely transcribed using PrimScript^TM^ RT reagent kit (TAKARA, USA) according to the instructions provided by the manufacturer. The real-time PCR reactions for the detection of mRNA were performed using the SYBR^®^ Premix Ex Taq™ II (TAKARA). All the primer sequences are listed in Additional file 1: Table S1. The levels of mRNA expression were normalized to GAPDH. The 2^−ΔΔCt^ method was used to quantify relative mRNA expression levels.

### Proliferation and apoptosis of CD4^+^ T cells

Following a 6-h transfection period, cells were analyzed for evidence of proliferation and apoptosis. For proliferation detection, CD4^+^ T cells were labeled with CellTrace™ Violet (5M; Life Technologies, Carlsbad, CA, USA) in PBS and incubated for 15 min at 37 °C. After washing in 1640 complete media supplemented with 10% FBS, cells were stimulated with soluble anti-CD3/anti-CD28 antibodies (1 μg/mL; BD Biosciences) and cultured in 96-well plates (200 μL) at 37 °C, 5% CO_2_, for 4 days. Dead cells were excluded by adding 7-aminoactinomycin D (7-AAD) to the culture medium prior to sample analysis. For apoptosis detection, CD4^+^ T cells were cultured for 2 days at 37 °C, 5% CO_2_. After the culture period, cells were stained with 5 μL 7-AAD and anti-Annexin V-PE for 15 min prior to data acquisition. Cells were acquired on the LSR II flow cytometer (BD Biosciences) and analyzed using Flowjo software (TreeStar).

### Statistical analysis

SPSS version 17.0 (SPSS Inc, USA) and Graphpad Prism (GraphPad, Ca) software were used to conduct statistical analyses. Paired t-test and wilcoxon matched-pairs signed rank test were used to assess differences between groups. Correlations between variables were evaluated using the Spearman rank correlation test. P values < 0.05 were statistically significant.

Negative binomial regression models were run for each set of comparisons with the percentage of CD38 Tcm subsets and the total HIV DNA. We chose this approach for reasons described previously [23, 31, 32]. Analyses were run using Stata software (Stata Corp, USA).

## Results

### Unlike HLA-DR, CD38 is highly expressed on CD4^+^ Tcm cells from HIV infected individuals on long-term ART

**Fig. 1:**
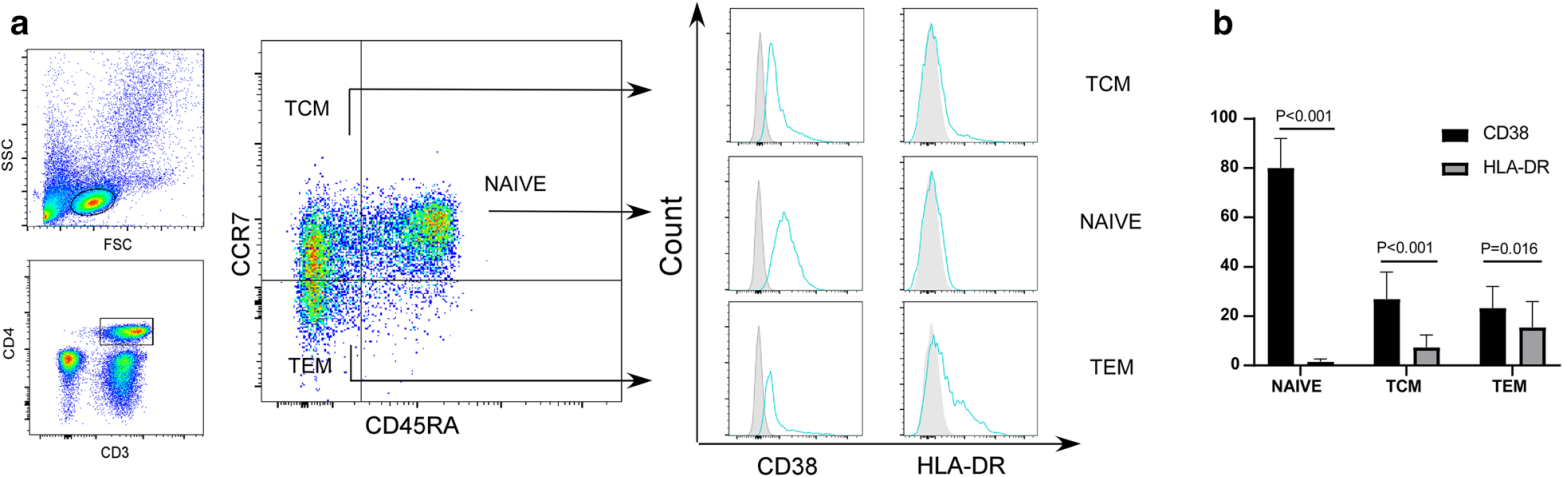
CD38 is highly expressed on the CD4^+^ Tcm cells of HIV-infected individuals on long-term ART. **a** FACS analysis of CD38 and HLA-DR expression on various CD4^+^ T cell subsets (naïve (Tn), central memory (Tcm)), and effector memory (Tem) in PBMCs obtained from HIV-infected individuals receiving ART for > 5 years. **b** Quantification of the percentages of CD38^+^ and HLA-DR^+^ CD4^+^ T cells associated with the Tn, Tcm, and Tem cell subsets. Wilcoxon matched-pairs signed rank test; n = 18 donors

Firstly, we studied the expression profiles of CD38 and HLA-DR on CD4^+^ Tcm, Tem and Tn cells in 18 HIV-infected subjects (cohort 1), receiving suppressive ART for a median time (Interquartile range, IQR) of 6.3 years (5.3–6.9) and a median CD4^+^ T cell count (IQR) of 487 cells/μl (377–884). PBMCs were isolated from the peripheral blood of HIV-infected subjects and analyzed by flow cytometry. We found that CD38, but not HLA-DR, was highly expressed on Tn, Tcm, and Tem cell subsets (P < 0.001, P < 0.001, and P = 0.016; Fig. 1a, b).

### CD4^+^CD38^+^ Tcm cells display lower levels of activation and higher levels of CD127 expression

**Fig. 2:**
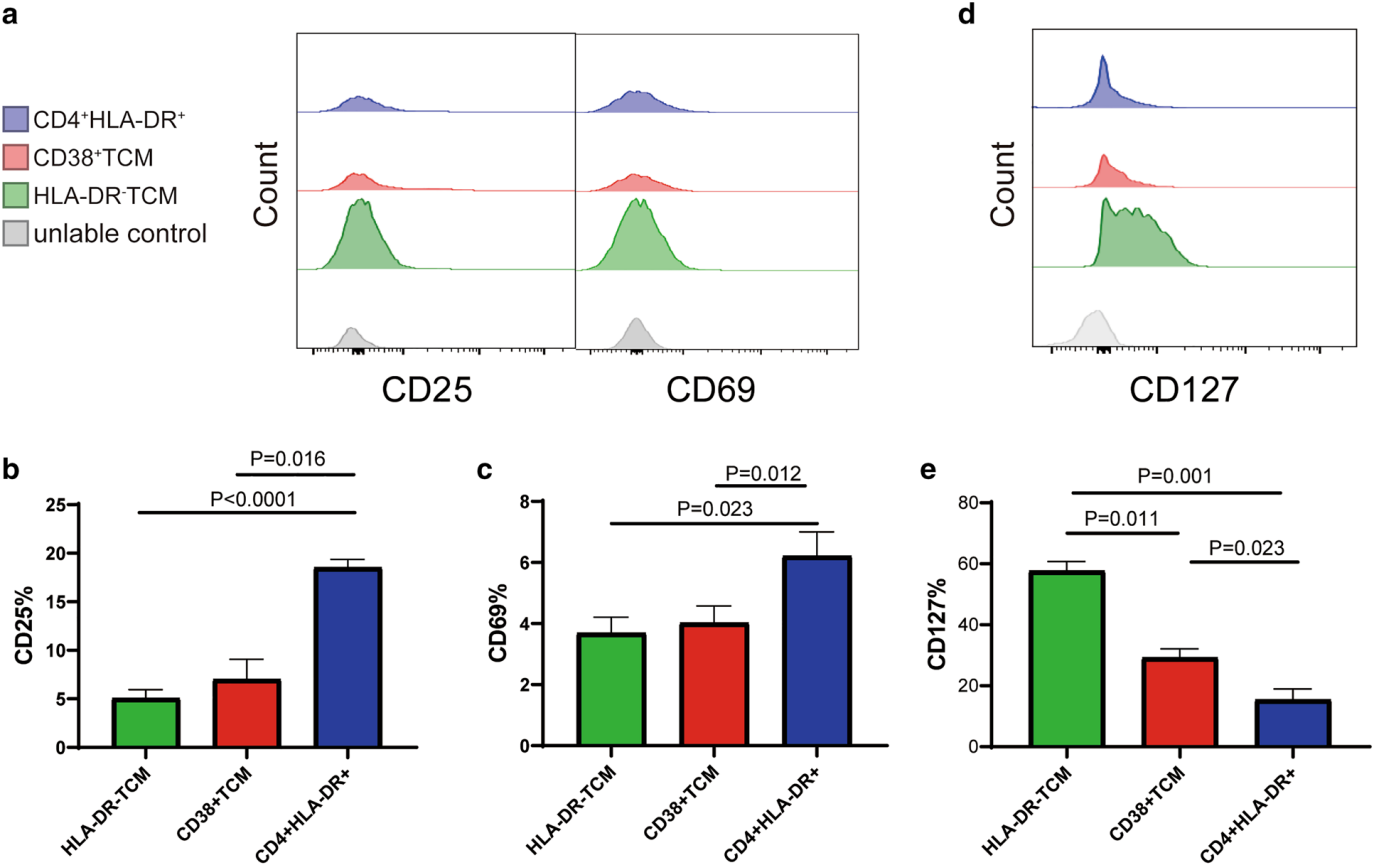
CD38^+^ Tcm is associated with lower activation and higher CD127 expression levels. **a** FACS analysis of CD25 and CD69 expression on CD4^+^CD38^+^ Tcm, CD4^+^HLA-DR-Tcm and CD4^+^HLA-DR^+^ T cells in PBMCs obtained from HIV-infected individuals receiving ART for > 5 years. Quantification of the percentages of CD25^+^ cells (**b**) and CD69^+^ (**c**) cells in CD4^+^CD38^+^ Tcm, CD4^+^HLA-DR^−^ Tcm, and CD4^+^HLA-DR^+^ T cell compartments. Paired t-test; data from 3 donors. **d** FACS analysis of CD127 expression on CD4^+^CD38^+^ Tcm, CD4^+^HLA-DR^−^ Tcm, and CD4^+^HLA-DR^+^ T cells with PBMCs obtained from HIV-infected individuals receiving ART for > 5 years. **e** Quantification of the percentages of CD127^+^ cells within the CD4^+^CD38^+^ Tcm, CD4^+^HLA-DR^−^ Tcm, and CD4^+^HLA-DR^+^ T cell populations. Paired t-test; n = 3 donors

Next, we assessed the markers on CD4^+^CD38^+^ Tcm cells, which have been previously reported to be associated with HIV reservoir maintenance. HIV reservoir cells are generally characterized by a low activation state (CD25^−^ and CD69^−^) [33–35]. In accordance, we found that the CD4^+^CD38^+^ Tcm cells expressed low levels of the activation molecules CD25 and CD69 in three patients from cohort 1, which is similar to the classical CD4^+^HLA-DR^−^ Tcm reservoir cells, and significantly lower than the activated CD4^+^HLA-DR^+^ cells (P = 0.016, P = 0.012, respectively; Fig. 2a–c).

Immune cells harboring the HIV reservoir are typically associated with prolonged survival and persist for several decades. The α chain of the interleukin-7 (IL-7) receptor (CD127) promotes HIV persistence by enhancing the proliferation and survival of Tcm cells during ART [36]. By assessing the CD127 expression on different CD4^+^ T cell subtypes, we found that the expression of CD127 on the CD38^+^ Tcm population was significantly higher than that on CD4^+^HLA-DR^+^ T cells (P = 0.023; Fig. 2d, e). Collectively, these findings demonstrate the potential of the CD38^+^ Tcm cells to contribute to the establishment and maintenance of HIV persistence.

### The proportion of CD38^+^ Tcm cells correlates with and predicts total HIV DNA in CD4^+^ T cells

**Fig. 3:**
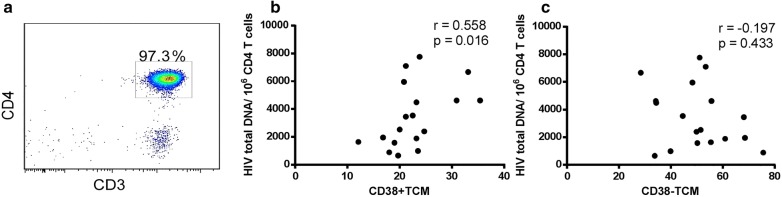
The proportion of CD38^+^ Tcm can predict total HIV DNA in CD4^+^ T cells. **a** Purity of CD4^+^ T cells sorted from PBMC obtained from HIV-infected individuals under at least 5 years ART. Numbers show percentages of CD4^+^ T cells. Correlations between total HIV DNA detected in CD4^+^ T cells and the percentage of CD4^+^CD38^+^ Tcm cells (**b**) or CD4^+^CD38^−^ Tcm cells (**c**). Spearman’s rank test; n = 18 donors

To determine the relationship between the proportion of CD38^+^ Tcm cells and total HIV DNA, CD4^+^ T cells were sorted from PBMCs of 18 HIV infected patients (cohort 1) and the total HIV DNA in the CD4^+^ T cells was detected by ddPCR. We found a significant positive correlation between the proportion of CD38^+^ Tcm cells (Tcm cells within the CD4^+^CD38^+^ T cell population) and total HIV DNA in CD4^+^ T cells (r = 0.558 and P = 0.016; Fig. 3b), while the proportion of CD38^−^ Tcm cells displayed no correlation with total HIV DNA (Fig. 3c). To further determine whether the proportion of CD38^+^ Tcm predicts total HIV DNA in CD4^+^ T cells, we used a negative binomial regression model, which can adjust for the current and nadir CD4^+^ T cell counts (Table 1). We found that the proportion of CD38^+^ Tcm cells can predict total HIV DNA in CD4^+^ T cells (P = 0.032). After correction with current CD4^+^ T cell or nadir CD4^+^ T cell counts, the predictable function of CD38^+^ Tcm to total HIV DNA still exists (P = 0.022 and P = 0.034; Table 1). These results indicate that the proportion of CD38^+^ Tcm can independently predict total HIV DNA in CD4^+^ T cells. The CD38^−^ Tcm subset did not show a significant correlation with total HIV DNA (Table 1), possibly indicating that CD38^+^ Tcm cells contribute more strongly to the maintenance of HIV persistence than CD38^−^ Tcm cells.

**Table 1:** Negative binomial regression models to assess the relationship between total HIV DNA and CD38^+/−^ Tcm expression on CD4^+^ T cells

Outcome	Predictor	Unadjusted		Adjusted for current CD4		Adjusted for nadir CD4	
		Result (95% CI)	P-value	Result (95% CI)	P-value	Result (95% CI)	P-value
Total HIV DNA	CD38^+^ Tcm	0.0596 (0.005 to 0.1142)	0.032	0.0615 (0.0087 to 0.1144)	0.022	0.0591 (0.0043 to 0.1139)	0.034
	CD38^−^ Tcm	− 0.0165 (− 0.0423 to 0.0093)	0.211	− 0.0188 (− 0.0449 to 0.0072)	0.156	− 0.0176 (− 0.0436 to 0.0085)	0.186

### CD38^+^ Tcm cells make a larger contribution to the viral reservoir than the CD38^−^Tcm population

**Fig. 4:**
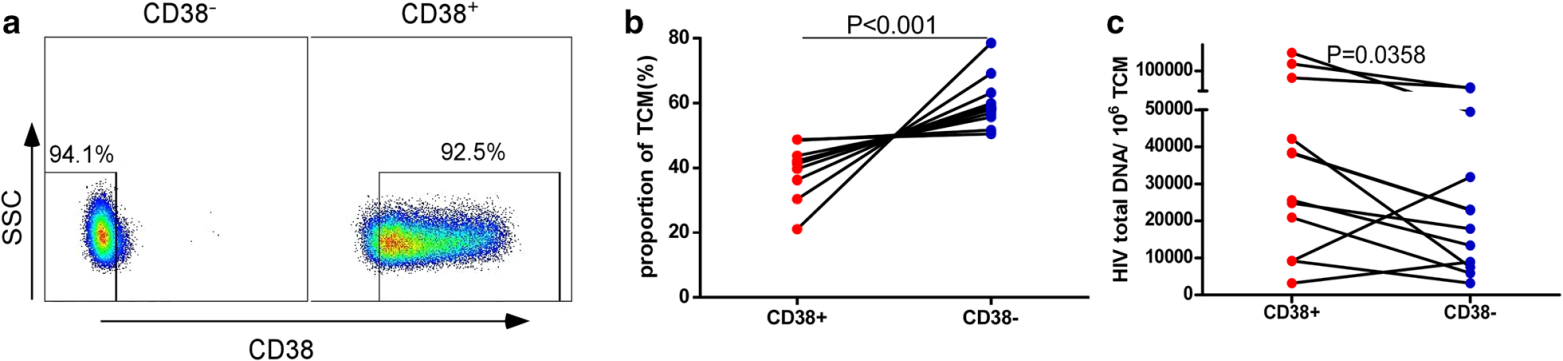
CD38^+^ Tcm cells make a larger contribution to the HIV reservoir than CD38^−^ Tcm cells. **a** Purity of CD38^−^ Tcm (left) and CD38^+^ Tcm (right) cells, sorted from PBMC obtained from HIV-infected individuals receiving ART for > 5 years. Numbers show percentages of CD38^−^ Tcm and CD38^+^ Tcm cells. **b** Quantification of the percentages of CD38^+^ and CD38^−^ Tcm subsets. Paired t-test; n = 12 donors. **c** Quantification of the total HIV DNA in CD38^+^ Tcm and CD38^−^Tcm cells. Paired t-test; n = 12 donors

To confirm whether the CD38^+^ Tcm cells contribute more to HIV persistence in Tcm, we sorted CD4^+^ Tcm cells, based on their expression of CD38, from 12 HIV-infected subjects (cohort 2) who had been on suppressive ART for a median time (IQR) of 5.5 years (5.3–6.9) and a median CD4^+^ T cell count (IQR) of 656 cells/μl (501–725). The purity of CD38^+^ Tcm and CD38^−^ Tcm populations were all > 90% (Fig. 4a). We subsequently measured total HIV DNA in these two groups by ddPCR. The results showed that although CD38^+^ cells accounted for a lower proportion of the Tcm population (P < 0.001; Fig. 4b), the CD38^+^ Tcm cells were associated with a higher total HIV DNA content than CD38^−^ Tcm cells (P = 0.0358; Fig. 4b). This analysis indicated that CD38^+^ Tcm made a larger contribution to the viral reservoir than the CD38^−^ Tcm population.

### CD38 promotes proliferation in the CD4^+^ T cells of HIV-infected individuals

**Fig. 5:**
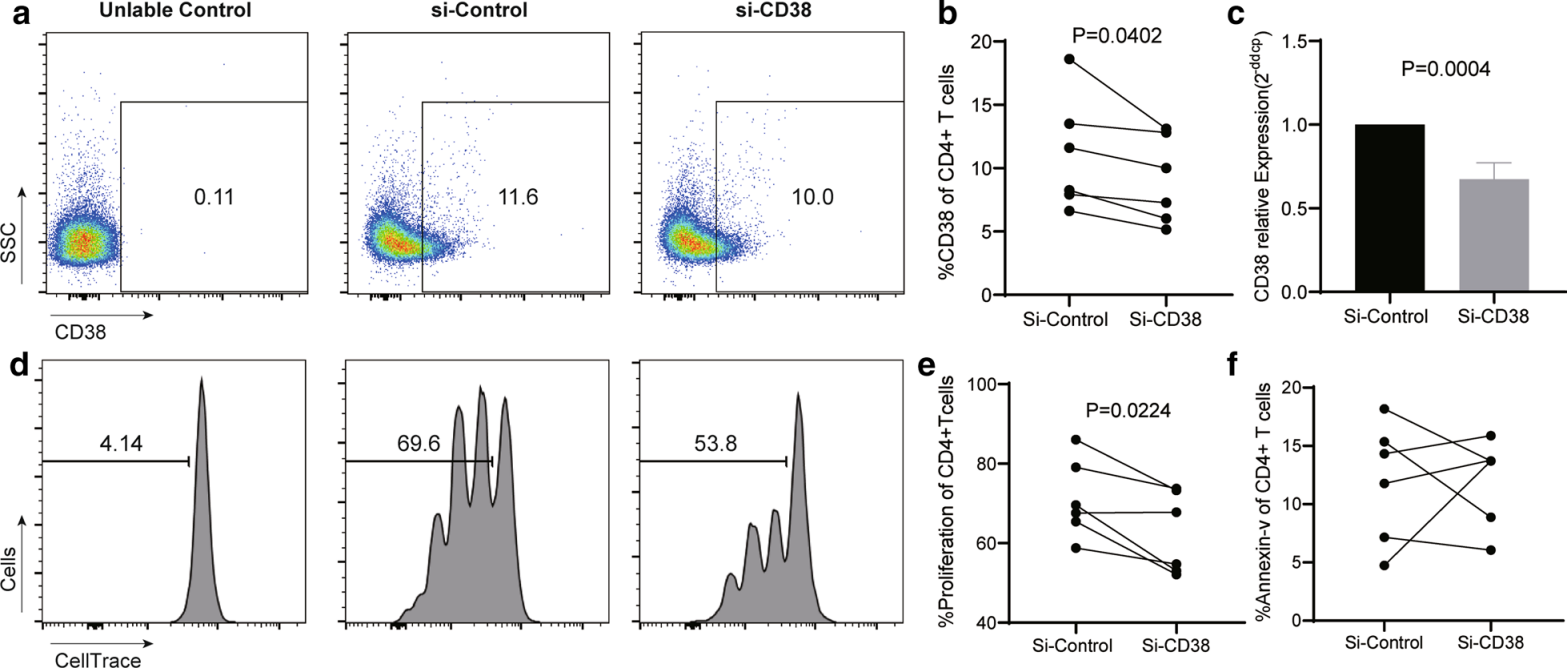
CD38 promotes proliferation in the CD4^+^ T cells of HIV-infected individuals. Expression of CD38 on CD4^+^ T cells after CD38 siRNA transfection. FACS analysis of CD38 expression on CD4^+^ T cells transfected with either negative control or CD38 siRNA for 72 h (**a**, **b**). **c** Real-time PCR of CD38 mRNA expression following a 48-h transfection period. FACS analysis of CD4^+^ T cell proliferation. **d**, **e** After a 6-h transfection period, CD4^+^ T cells were labeled with CELL Trace^TM^ Violet and stimulated using anti-CD3/CD28 antibodies (1 μg/ml) for 4 days. The proliferation of CD4^+^ T cells was determined. FACS analysis of CD4^+^ T cell apoptosis **f**. After a 48-h transfection period, CD4^+^ T cells were labeled with 7-AAD and Annexin-V, to quantify CD4^+^ T cell apoptosis. Wilcoxon matched-pairs signed rank test; n = 6 donors

Latently infected CD4^+^ T cells are maintained by homeostatic proliferation and survival mechanisms [11]. We have found that CD38^+^ Tcm cells express higher CD127 levels, which promote T cell survival and proliferation [37, 38]. To further explore the mechanistic basis for the contribution of CD38 to HIV persistence after long-term ART, we sorted CD4^+^ T cells from the PBMCs of 6 HIV-infected individuals (cohort 3) on suppressive ART for a median time (range) of 2 years (1.9–3.9) and a median CD4^+^ T cell count (range) of 525 cells/μl (406–1211). The CD4^+^ T cells were then transfected with 20uM of either CD38 siRNA or negative control siRNA and cultured for 24 h. Compared to negative control siRNA-transfected cells, CD4^+^ T cells transfected with the CD38 siRNA, significantly downregulated their CD38 expression (Figure 5a–c). To detect the proliferation of CD4^+^ T cells, transfected cells were stimulated with anti-CD3/CD28 antibodies (1 μg/ml) for 4 days. We found that the proliferation of CD38 siRNA-transfected CD4^+^ T cells was reduced, compared to the negative control (P = 0.0224; Fig. 5d, e). Then we tested the level of apoptosis after 2 days of transfection and we did not find a significant difference in the level of apoptosis between the two groups (Fig. 5f). Thus, CD38 may contribute to the maintenance of HIV persistence by promoting proliferation in the CD4^+^ Tcm subset.

## Discussion

Numerous studies have shown that despite the effective antiviral treatment of HIV-infected patients, complete viral eradication has not been achieved due to the persistence of the HIV reservoir [39–41]. Long-lived CD4^+^ Tcm cells represent important sites for HIV reservoir concealment [40, 42, 43]. In this study, we found that CD4^+^CD38^+^ Tcm contribute to HIV persistence in HIV-infected individuals receiving long-term ART, proposing novel strategies for HIV reservoir eradication.

Firstly, we found that unlike HLA-DR, CD38 is higher expressed on CD4^+^ Tcm in HIV-infected individuals under long-term ART. CD38 expression on CD8^+^ T cells is considered as an activation marker in HIV infection [12, 44, 45]. In children infected with HIV during the perinatal period, CD4^+^CD38^+^ subsets are in contrast, immature cells [17]. Moreover, in B-cell CLL, CD38 is also expressed on CD4^+^ Tn and Tcm subsets [18], and the proportion of CD38^+^ B cells is a predictor of clinical outcome [46]. Our study confirmed that CD38 is highly expressed in the CD4^+^ Tn and Tcm cells of HIV-infected individuals. We then found CD38^+^ Tcm expressed lower levels of the activation markers CD25 and CD69, but high levels of CD127. Due to its quiescent nature and prolonged survival, the Tcm subset of CD4^+^ T cells is the prime site for the HIV reservoir [47, 48], sustaining HIV replication through low-level antigen driven proliferation and IL-7 signaling. According to our findings, the low activation status and high CD127 expression demonstrated by CD4^+^CD38^+^ Tcm cells may imply that these cells belong to the group of homeostatic proliferating cell subsets [36, 37, 49].

Secondly, we found that CD4^+^CD38^+^ Tcm cells contribute to HIV persistence in HIV-infected individuals receiving long-term ART and can predict total HIV DNA in CD4^+^ T cells. Total HIV DNA and integrated DNA have long been recognized as important markers for detecting HIV reservoir cells [11, 50]. We sorted CD4^+^ T cells from HIV-infected subjects who had undergone > 5 years of suppressive ART, and found that the proportion of CD38^+^ Tcm cells positively correlated with total HIV DNA in the CD4^+^ T cells. Furthermore, the proportion of CD38^+^ Tcm, but not CD38^−^ Tcm cells, can predict the total HIV DNA of CD4^+^ T cells. In contrast, Murray et al. showed that there was no difference in the total HIV DNA between the CD45RO^+^CD38^+^ and CD45RO^+^CD38^−^ T cell subsets of HIV-infected individuals after a year on ART. However, the same study also found that the virus in CD38^+^ memory T cells had a longer half-life than in CD38^−^HLA-DR^−^ memory T cells [30]. Since the virus in CD38^+^ Tcm cells has a longer half-life, compared to in the CD38^−^ Tcm population, these HIV reservoirs may be easier to maintain following long-term ART [27–29], explaining why we found more HIV DNA within the CD38^+^ than the CD38^−^ Tcm compartment, after long-term ART. Our further study confirmed that although the proportion of CD38^+^ cells in Tcm was lower than CD38-cells, they harbored higher levels of total HIV DNA compared to CD38^−^ Tcm cells, suggesting that the CD38^+^ subset is more importance to the HIV persistence in Tcm. Our results were consistent with Pallikkuth et al.’ study [51]. They found that peripheral T follicular helper cells (pTfh), a subset of CD4^+^ Tcm cells, are highly susceptible to HIV infection. Compared with non-pTfh cells, pTfh cells highly express CD38 and HIV persists in these cells following plasma virus suppression with potent cART. These data suggest that high CD38 expression are helpful to HIV persistence.

Finally, we demonstrate that CD38 expression promotes the proliferation of CD4^+^ T cells derived from HIV-infected patients undergoing long-term ART. The Tcm reservoir is one of the most significant HIV reservoirs. Due to its homeostatic proliferation and prolonged lifespan, the HIV reservoir can remain stable over time [40]. The role of CD38 in cell proliferation and apoptosis differs between diseases. Liao et al. found that CD38 can promote proliferation and inhibit apoptosis in cervical cancer cells [52]. In CLL cells, CD38/CD31 interactions enhance cell proliferation and migration by activating various genetic pathways [22]. In sepsis-related brain damage in rats, however, the CD38/cADPR pathway may promote apoptosis [53]. We found that CD38 expression on CD4^+^ T cells enhanced cell proliferation but has no effect on apoptosis in HIV-infected individuals, indicating that CD38 may contribute to viral persistence by promoting the homeostatic proliferation and prolonging the lifespan of CD4^+^ Tcm cells. There are many small molecule antagonists of CD38 have been developed [54–56] and daratumumab (human IgGκ monoclonal antibody that targets CD38) have been used clinically to treat multiple myeloma and achieved good results [57]. According to our results, it provides an important basis for the application of effective CD38 small molecule antagonists to inhibit HIV persistence.

## Conclusions

In summary, our study found that CD38 contributes to HIV persistence by enhancing the proliferation of Tcm cells in HIV-infected individuals undergoing long-term ART. Our findings provide a partial explanation for why HIV reservoir eradication is not achieved following long-term ART, as well as propose new strategies for suppressing HIV persistence. In recent years, many small molecule CD38 antagonists have been developed [54–56]. For instance, daratumumab (anti-CD38 IgGκ mAb) has been successful in treating multiple myeloma in the clinic [57]. Our results, therefore, provide a basis for the application of CD38-targeting antagonists to resolve HIV persistence.

## Supplementary information

**Additional file 1: Table S1.** Primers used for ddPCR and RT-PCR.

## Data Availability

The authors can confirm that all relevant data and materials are available on request from the authors.

## Abbreviations

HIV: Human immunodeficiency virus
ART: Antiretroviral treatment
AIDS: Acquired immune deficiency syndrome
Tn: Naive T cells
Tcm: Central memory T cells
Tem: Effector memory T cells
CLL: Chronic lymphocytic leukemia
PBMC: Peripheral blood mononuclear cell
mRNA: Message RNA
FMO: Fluorescence minus one
RT-PCR: Real-time PCR
ddPCR: Digital droplet PCR
