# Supplementary material for: CD4+CD38+ central memory T cells contribute to HIV persistence in HIV-infected individuals on long-term ART
Source: J Transl Med. 2020 Feb 24;18:95. doi: 10.1186/s12967-020-02245-8 (PMC7038621; doi:10.1186/s12967-020-02245-8)
Supplement: Supplementary file 1 — Additional file 1: Table S1. Primers used for ddPCR and RT-PCR. [file 12967_2020_2245_MOESM1_ESM.docx]

**Table S1. Primers used for ddPCR and RT-PCR**

| **symbol** | **sequence (5'-3')** |
| --- | --- |
| LTRG-F | TACTGACGCTCTCGCACC |
| LTRG-R | TCTCGACGCAGGACTCG |
| LTRG-P | CTCTCTCCTTCTAGCCTC |
| RPP30-F | GATTTGGACCTGCGAGCG |
| RPP30-R | GCGGCTGTCTCCACAAGT |
| RPP30-P | CTGACCTGAAGGCTCT |
| GAPDH-F | CGACCACTTTGTCAAGCTCA |
| GAPDH-R | ACTGAGTGTGGCAGGGACTC |
| CD38-F | TGCTGATGACCTCACATGGT |
| CD38-R | CCATTGAGCATCACATGGAC |
